# A pilot randomized controlled trial comparing CABG surgery performed with total arterial grafts or without

**DOI:** 10.1186/s13019-014-0203-8

**Published:** 2015-01-08

**Authors:** Jeffrey Le, Roger JF Baskett, Karen J Buth, Gregory M Hirsch, Allan Brydie, Ryan Gayner, Jean-Francois Legare

**Affiliations:** Department of Surgery, Dalhousie University, Halifax, Nova Scotia Canada; Department of Radiology, Dalhousie University, Halifax, Nova Scotia Canada; Division of Cardiovascular Surgery, The Maritime Heart Center, 2269-1796 Summer Street, Halifax, NS B3H 3A7 Canada

**Keywords:** CABG artery, Arteries, CABG, Outcomes

## Abstract

**Objective:**

To date only a few randomized controlled studies have compared grafting strategies in patients with multi-vessel coronary disease. This study represents a pilot RCT designed to test the feasibility of a trial comparing conventional CABG performed with a LIMA-LAD plus saphenous vein grafts (LIMA+SVG) and CABG performed with total arterial grafting (TAG).

**Methods:**

Consenting patients undergoing non-redo isolated CABG surgery at a single institution were randomized to TAG or LIMA+SVG groups. Exclusion criteria included prior CABG, emergent procedure, concomitant procedure, varicose veins and renal dysfunction. The primary endpoints were: enrolment >20% and completion of CT coronary angiography at 6 months >80%. Statistical investigation was performed on an intention to treat analysis.

**Results:**

Of 421 eligible patients, 60 were enrolled and 2 withdrew (n = 30 in TAG, n = 28 LIMA+SVG) for 14% enrolment rate. Patient characteristics were similar in each group. No patients died in hospital and adverse events such as MI, stroke and deep sternal wound infection were not significantly different between groups. Clinical follow-up was complete in 100% of patients, with 44/58 (76%) undergoing CT coronary angio at 6 months. Graft occlusion occurred in 2 patients in each group for patency rates of 89% (TAG) and 91% (LIMA+SVG).

**Conclusions:**

We provide evidence that an RCT comparing grafting strategy is possible but also show that achieving recruitment or follow-up CT may be difficult. Given the excellent patency results and little difference between groups, our findings suggest that the sample size required may make it infeasible to compare graft patency at 6 months as a study end-point.

**Trial registration:**

Randomized Controlled Trial number: ISRCTN80270323.

**Ultra-mini abstract:**

Few RCT’s exist comparing conventional CABG performed with a LIMA-LAD plus saphenous vein grafts (LIMA+SVG) compared to CABG performed with total arterial grafting (TAG). This study is a pilot RCT designed to test the feasibility of such a trial and identify pitfalls.

## Background

Grafting of the left internal mammary artery (LIMA) to left anterior descending artery (LAD) is the established standard of care during coronary artery bypass graft (CABG) surgery [1]. However, grafting strategies to territories other than the LAD, involving the use of either arterial grafts versus saphenous vein(s) is still a matter of debate. This in spite of evidence suggesting superior patency and better long-term clinical outcome with arterial grafts [2-5]. This is reflected in the overall use of total arterial grafts, which remains very low, with less than 5% in patients within the Society of Thoracic Surgery (STS) Adult Cardiac Surgery database receiving multiple arterial grafts [6].

Most of the evidence favoring multiple arterial grafts has been obtained from retrospective analysis of large registry data [2-5]. These studies suggest that the use of multiple arterial grafts may improve long-term survival after CABG. A limited number of randomized controlled trials have been published examining all arterial grafting strategies without consistent findings. In a randomized controlled trial of 160 patients, Muneretto et al., demonstrated superior graft patency and lower return of angina in TAG patients [7]. In contrast, a randomized controlled trial involving 331 patients, Damgaard et al., was unable to show any significant differences in patency or clinical endpoints between TAG vs. LIMA+SVG grafting strategies at one year follow up [8]. Of note the primary outcomes for the study from Damgaard et al. were graft patency and cardiac event –free survival at 1 year.

The above reported randomized study highlight conflicting evidence in the literature and the relative paucity of definitive large randomized studies comparing total arterial grafting strategy (TAG) to saphenous vein and arterial grafting strategy. We sought in the present trial to test the feasibility of conducting such a trial by performing first a small-scale pilot trial in which barriers would be explored including taking into consideration individual surgeon practice as part of our analysis. In the present study we will demonstrate 3 important barriers to consider in designing a definitive trial comparing grafting strategy with TAG versus LIMA+SVG and how these can impact feasibility.

## Methods

### Randomized control trial study design

Given the paucity of randomized evidence available to guide surgeons in choosing the best grafting strategy for their patients, the present study was designed as a pilot prospective randomized controlled trial comparing total arterial grafting (TAG) to conventional grafting using a LIMA to the LAD and saphenous vein grafts for the other territories (LIMA+SVG) in patients undergoing non-redo isolated CABG.

Patients were excluded from enrolment if they were scheduled to undergo emergent surgery, single vessel bypass surgery, had received a prior CABG or displayed evidence of varicose veins on pre-operative physical examination. In addition, patients with a documented history of chest radiotherapy, allergy to contrast media and renal insufficiency (defined by serum creatinine > 176 μmol/L), were also excluded.

Informed consent was obtained from all patients in accordance with our institutional research ethics review board guidelines (Randomized Controlled Trial number: ISRCTN80270323). Once the patient had consented, he or she was randomized prior to entering the operating room. The randomization process was performed using random permuted blocks with varying block sizes of 4, 6 and 8. Numbered index cards were placed in sealed, opaque envelopes and stored safely with the research coordinator. Consented patients were randomized prior to surgery, and the surgeon opened the envelope in the operating room before starting the case. The index card indicated whether the patient was to be randomized to TAG or LIMA+SVG. If the surgeon decided intra-operatively that the patient could not undergo the procedure to which he or she was randomized, the patient was offered the revascularization strategy most likely to afford the patient the best possible outcome. If the patient required a concomitant cardiac procedure (e.g. valve replacement or repair) or could not undergo a revascularization procedure with a minimum of two distal bypasses, the patient was excluded from the study.

### Primary outcome

The primary objectives of our pilot study were to evaluate the feasibility of a larger randomized trial in current cardiac surgical practice in which individual surgeon preferences have been suggested to play a large role in choosing grafting strategies [1]. Feasibility was defined as our ability to recruit more than 20% of eligible patients and assess completion of CT-coronary angiography in greater than 80% of patients at 6 month follow-up.

### Secondary outcome

The secondary objectives of the study were to compare postoperative graft patency and in-hospital mortality and morbidity. In addition the quality of graft imaging was also examined. All analysis was performed on an intent-to-treat basis. The treating team and patients were not blinded to the treatment allocation. However, CT-coronary angiograms were reviewed in a blinded manner by a single radiologist.

### Operative technique

All interventions were performed via a midline sternotomy, and cardiopulmonary bypass was utilized in a standardized manner for all cases. Body temperature during the procedure was allowed to drift briefly to 32°C. Intermittent cold blood cardioplegia solution was delivered anterograde via the aortic root unless otherwise indicated. Arterial conduits were harvested with minimal trauma (LIMA and RIMA were not skeletonized) and were treated with either a papaverine solution or a nitroglycerine/calcium channel blocker (verapamil) solution prior to their use. TAG was defined as the use of any arterial conduit (LIMA, RIMA or radial artery), either alone or in combination, without concomitant use of SVG. The choice of conduit and the manner in which the grafts were constructed, including composite T- or Y-grafts, proximal aorto-coronary anastomoses or sequential anastomoses, was based entirely on surgeon preference rather than on any fixed criteria such as territory to graft or degree of target vessel stenosis. LIMA+SVG was defined as any case in which the LIMA was used for a single bypass to the LAD and SVGs were used for the remaining bypasses, constructed as either a series of sequential anastomoses or as single bypasses and were anastomosed proximally to the aorta.

### Post- operative management

All study patients received postoperative intravenous nitroglycerine infusions for the first 24 hours upon return from the operating room unless hypotensive (systolic blood pressure < 90 mm Hg). Other routine post-operative medications included daily aspirin as well as resumption of cholesterol lowering agents, B-blockers and angiotensin converting enzyme inhibitors as appropriate.

### CT-coronary angiography

All study patients received CT angiography 6 months post CABG. This was performed on a 64 slice multi-detector CT scanner (Siemens Sensation 64, Erlangen, Germany), using the following scan parameters: 330 ms gantry rotation, detector collimation 32 × 0.6 mm (with a rapidly alternating focal spot resulting the acquisition of 64 slices per gantry rotation with effective special resolution of 0.4 mm), tube voltage 120 kV, maximum obtainable tube current (800 – 900 mAs) scanning in a caudo-cranial direction. Retrospecive ECG gating was used with ECG pulsing such that full tube current was applied between 50% and 80% of the cardiac cycle, and reduced to 40% during the remainder. One hundred mL of contrast agent (Isovue 370, Bracco, Italy) was injected into a right antecubital vein via an 18-guage cannula at a flow rate of 5 mL/s, followed by 40 mL of normal saline bolus chaser at a rate of 5 mL/s. All patients with heart rate >65 beats per minute, unless contra-indicated, received beta blocker in the form of 50 mg oral metoprolol, 0.2 micrograms of sublingual glycerol trinitrate, plus or minus 1–2 mg oral lorazepam prior to being scanned in order to reduce heart rate and maximize the lumen diameter of the coronary artery bypass grafts. Axial data sets with a slice thickness 0.75 mm and slice interval 0.6 mm, were reconstructed using a medium smooth reconstruction kernel (B30f) at the following intervals during the cardiac cycle (20%, 55%, 60%, 65%, 70%, 75%). Further datasets from different time intervals or at a sharper reconstruction kernel (B46f) were reconstructed at the interpreting radiologist’s discretion. Interpretation of all CT angiograms was performed by a single level 3 trained cardiac radiologist who was blinded to the treatment allocation.

Each bypass graft was assessed for image quality with graft visualization determined as excellent, diagnostic or non-diagnostic. The presence or absence of artifact was noted with the following categories: none, clip, rhythm, respiratory motion and other. Heart rate and heart rhythm during the CTA acquisition were recorded.

Each bypass graft for which visualization was determined not to be non-diagnostic was visually assessed using axial data, multi-planar reformats and curved planar reformats as per Society of Cardiovascular Computed Tomography guidelines and assigned to one of three categories: Occluded (defined as any focus where there was complete lack of luminal contrast), luminal stenosis of greater than 50%, or patent (no luminal stenosis greater than 50%) [9].

### Data sources

The Maritime Heart Center (MHC) Cardiac Surgery Registry captures pre-operative, intra-operative and in-hospital post-operative clinical variables including mortality and morbidity for all patients undergoing cardiac surgery at the QEII Health Sciences Centre in Nova Scotia, Canada. Pre-operative variables included age, sex, body mass index (BMI), smoking history, diabetes, hypercholesterolemia, hypertension, peripheral and/or cerebrovascular disease, chronic obstructive pulmonary disease, left ventricular ejection fraction (EF), myocardial infarction within 21 days prior to surgery, urgency of surgery (urgent patients required surgery within 24 hours, in-house patients were waiting in-hospital, elective patients were waiting at home), prior percutaneous coronary intervention, left main stenosis >50%, and number of diseased vessels. In-hospital outcomes included all-cause mortality, stroke (neurological deficit persisting at time of hospital discharge), peak troponin T, deep sternal wound infection, mechanical ventilation >24 hours, and postoperative length of stay in hospital >9 days. Aortic cross-clamp time was also reported.

### Statistical analysis

The TAG and LIMA+SVG groups were compared using Chi-square or Fisher’s exact tests for categorical variables, two-tailed t-tests for continuous variables that were normally distributed, and Wilcoxon rank sum tests for continuous variables that did not have a normal distribution.

One of the primary objectives was > 20% recruitment of patients into the randomized trial. Patient eligibility was based on inclusion and exclusion criteria and assignment to participating surgeons. This allowed for the creation of a predictive model capable to identify factors that predicted use of TAG. Design variables were created for reference level coding of categorical variables with more than two levels. For the multivariable analysis, candidate variables were selected based on clinical relevance or a significance of bivariate association with p value <0.2. A non-parsimonious logistic regression model was developed to identify the predictors of receiving TAG. The area under the receiver operating characteristic (ROC) curve was used to assess predictive accuracy of the model. A bootstrap procedure was used to obtain 1000 subsamples with replacement. The 2.5th and 97.5th percentiles of the bootstrap distribution were then used to determine the 95% Confidence Interval (CI) of the ROC.

All statistical analyses were performed using the SAS software package version 9.2 (SAS, Cary, North Carolina).

## Results

### Study population

During the study period, 421 patients met the eligibility criteria for participation in the trial. From this group of eligible patients, a total of 60 consented patients were randomized into 2 groups: TAG or LIMA+SVG. Two patients withdrew from the study leaving 58 patients (TAG: n = 30, LIMA+SVG: n = 28). The two study groups did not differ significantly in age, sex, EF, triple vessel disease, or other preoperative clinical characteristics (Table 1).

**Table 1:** **Pre-operative characteristics of TAG and LIMA+SVG groups**

	**TAG n = 30**	**LIMA+SVG n = 28**	**P value**
Age ≥ 70	4 (13)	5 (18)	0.73
Female	1 (3)	1 (4)	0.99
BMI < 25 kg/m^2^	3 (10)	4 (14)	0.70
Smoking history	24 (80)	20 (71)	0.45
Diabetes mellitus	9 (30)	6 (21)	0.46
Hypercholesterolemia	25 (83)	27 (96)	0.20
Hypertension	22 (73)	23 (82)	0.42
Myocardial infarction ≤ 21 days	2 (7)	4 (14)	0.42
EF < 40%	1 (3)	0 (0)	0.99
PVD and/or CVD	3 (10)	2 (7)	0.99
COPD	2 (7)	3 (11)	0.67
Left main stenosis > 50%	9 (30)	7 (25)	0.67
Triple vessel disease	20 (67)	16 (57)	0.46
Prior PCI	3 (10)	3 (11)	0.99
Urgency of surgery:			0.39
Elective	22 (73)	18 (64)	
In-house	7 (23)	10 (36)	
Urgent < 24 hours	1 (3)	0 (0)	

Categorical variables are shown as number (percent).

TAG: total arterial grafting; LIMA: left internal mammary artery; SVG: saphenous vein graft; BMI: body mass index; EF: left ventricular ejection fraction; CVD: cerebrovascular disease; PVD: peripheral vascular disease; COPD: chronic obstructive pulmonary disease; PCI: percutaneous coronary intervention.

Unadjusted in-hospital outcomes are shown in Table 2. There was no in-hospital mortality in either TAG or LIMA+SVG group. Furthermore no statistically significant differences were detected in stroke, deep sternal wound infection, mechanical ventilation >24 hours or postoperative length of stay >9 days. Peak troponin T and aortic cross-clamp time were significantly higher in the TAG group.

**Table 2:** **Unadjusted postoperative in-hospital outcomes**

	**TAG n = 30**	**LIMA+SVG n = 28**	**P value**
All cause mortality	0 (0)	0 (0)	-
Stroke	2 (7)	0 (0)	0.49
Deep sternal wound infection	0 (0)	0 (0)	-
Mechanical ventilation > 24 hours	3 (10)	0 (0)	0.24
Postoperative length of stay > 9 days	4 (13)	1 (4)	0.35
Peak troponin T, ng/mL	0.38 (0.21 – 0.55)	0.22 (0.13 – 0.30)	0.0051
Aortic cross-clamp time, minutes	86 (61 – 110)	56 (44 – 66)	0.0046

Categorical variables are shown as number (percent), and continuous variables are shown as median (inter-quartile range).

TAG: total arterial grafting; LIMA: left internal mammary artery; SVG: saphenous vein graft.

### Primary endpoints of the trial

**Figure 1:**
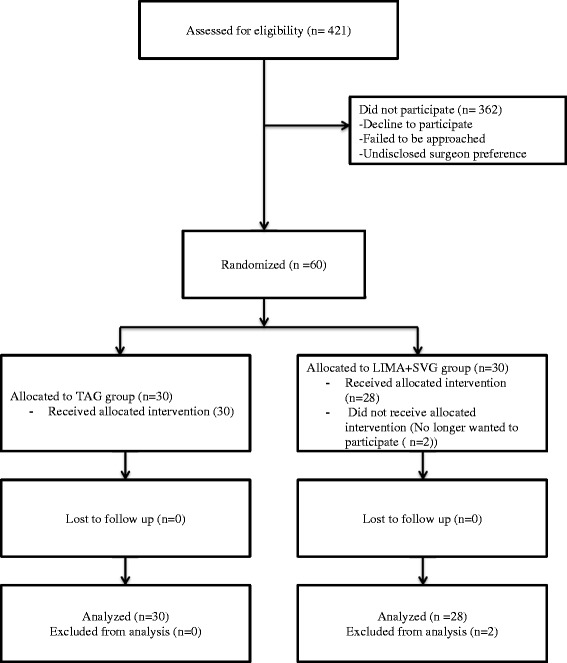
**Flow diagram primary outcome.**

The primary objectives were to recruit > 20% of eligible patients and to complete 6-month follow-up CT-coronary angiography in > 80% of study patients. The primary objectives of this study were not achieved with only 14.3% of eligible patients recruited (60/421) and 76% (44/58) of patients completing CT angiography. Of the 58 patients in the study clinical follow-up was complete in 100% of patients (Figure 1).

### Graft patency assessed by CT angiography

A total of 44 patients underwent CT angiography (23:TAG and 21: LIMA+SVG). Graft occlusion was observed in 2 patients (9%) in the TAG group and 2 patients (10%) in the LIMA+SVG groups (p = 0.99). Significant graft abnormality defined as graft occlusion or stenosis > 50% was present in 3 patients (13%) in TAG group and 3 patients (14%) in LIMA+SVG groups p = 0.99). We then compared the patency at 6 months between arterial grafts and saphenous vein grafts outside of their group allocation. There were a total of 83 arterial grafts compared to 34 saphenous grafts. In this analysis (n = 117 grafts), CT angiography demonstrated that graft patency was greater than 95% in both groups (p = 0.99).

The quality of images was also analyzed as an endpoint in this pilot trial. It should be noted that visualization of 7 out of 117 grafts (6%) was determined to be non-diagnostic. This was observed in 5 patients in TAG group compared to 1 patient in LIMA+SVG group (p = ns). Four of the six non-diagnostic grafts were sequential grafts to the lateral wall of the left ventricle when compared to free radial graft to the inferior or lateral wall. The most common reason for difficulty visualizing was clip artifact. Details on patients and grafts that were non-diagnostic are illustrated in Table 3.

**Table 3:** **Features of patients in which grafts visualization was difficult**

	**Group**	**Reason for poor visualization**	**HR and Rhythm**	**Type of graft**
#1	TAG	Clip artifact	NSR, HR < 65	Sequential RIMA to OM and PDA
#2	TAG	Clip and rhythm artifact	NSR, HR < 63	Sequential RIMA to OM1 and OM2
#3	TAG	Clip and respiratory artifact	NSR, HR < 74	Free radial to OM1
#4	TAG	Clip and rhythm artifact	NSR, HR < 84	Free radial to PDA
#5	TAG	Clip artifact	NSR, HR < 69	Sequential graft to OM and LVb
#6	LIMA+SVG	Clip artifact	NSR, HR < 68	Sequential graft SVG to LVb and PDA

HR: heart rate; TAG: total arterial grafting; NSR: normal sinus rhythm; RIMA: right internal mammary artery; OM: obtuse marginal branch of the circumflex; PDA: posterior descending artery; LVb: left ventricular branch of the circumflex; SVG: saphenous vein graft.

### Predictors of total arterial grafting

**Figure 2:**
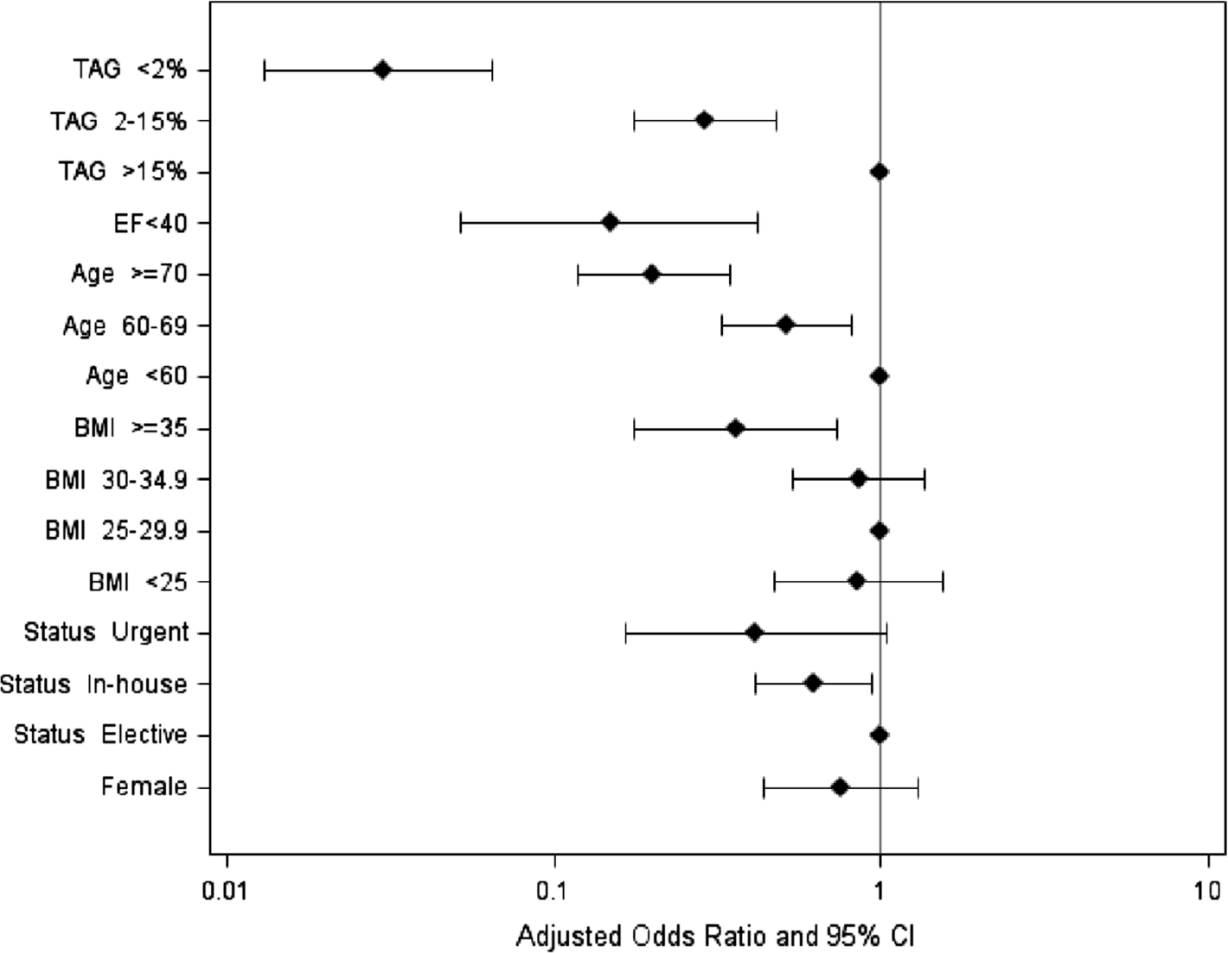
**Plot of adjusted odds ratios and 95% confidence intervals for predictors of total arterial grafting.** TAG: total arterial grafting; EF: left ventricular ejection fraction; BMI: body mass index.

We created a non-parsimonious logistic regression model predicting the probability of a CABG patient receiving TAG during the entire study period in order to better understand variables responsible for patient selection. The following variables were included in the model: surgical volume (proportion of TAG cases performed by each surgeon), age, sex, BMI, EF and urgency of surgery. A plot of the variables with adjusted odds ratios is shown in Figure 2. The model had good predictive accuracy with an ROC of 86% and 95% confidence interval 83% - 90%. The strongest predictor was surgeon volume in performing TAG, independent of other clinically relevant variables such as age, sex, EF, BMI and urgency of surgery. Our findings would suggest that only surgeons who perform TAG more than 15% of the time are most likely to perform TAG and as such likely to enroll patients in this type of trial.

## Discussion

Numerous studies based on registry data have suggested that multiple arterial grafting in CABG patients provides a survival benefit over conventional CABG performed with a LIMA graft to the LAD and saphenous veins [2-5]. However, there have been few randomized studies have addressed this question [7,8]. In fact, this paucity of high-level evidence supporting the use of multiple arterial grafts may be responsible at least in part to the limited adoption of multiple arterial grafts. This lack of adoption is best illustrated by the fact that less than 5% of CABG patients in the STS database receive multiple arterial grafts [6].

The objective of the present pilot study was to look at the feasibility of conducting such a randomized study comparing grafting strategies. We defined feasibility as the ability to recruit a reasonable proportion of patients and to ensure that the majority would complete the follow-up. The recruitment was set at 20% of eligible patients, which represents a conservative estimate so that findings from the larger study could be applied to a modern CABG practice rather than highly selected cases. Study follow-up was defined as completion of a CT-angiogram at 6 month after surgery in more than 80% of patients. In this pilot study we failed to meet the primary objective with a recruitment of 14.3% of eligible patients and completion of CT angiography in 76% of patients. Our findings suggest therefore that a primary trial comparing TAG versus conventional CABG may be plagued by poor recruitment, which could compromise the generalizability of the findings.

However, the present study did demonstrate that in selected patients, CABG surgery can be safely performed with both grafting strategies ie. TAG or LIMA+SVG. In the 58 patients in the study there was no in-hospital mortality. However, it should be noted that TAG patients required longer clamp times and had significant higher Troponin T levels post-operatively. This findings is of importance given evidence that increased incidence of myocardial injury has been shown to impact outcomes after surgery [10,11]. While we reported in our study no new q wave myocardial infarction, our findings suggest that TAG patients may be at higher risk for myocardial injury assessed by a sensitive Troponin assay. We speculate that the Troponin rise is likely multifactorial but could easily be related to the longer cross-clamp times. Our observed longer cross clamp time highlights inherent higher technical challenges in using only arterial grafts compared to their venous counterparts which in our study translated in longer myocardial exposure to ischemia. The methods used for myocardial protection were identical between groups suggesting that our observation is unlikely related to inadequate myocardial protection.

In the patients that had follow-up CT angiography (76% of patients), graft patency in both groups of patients was found to be greater than 90%. Our findings of similar 6-month patency between groups would suggest that a very large study would be required to be able to detect a patency difference between groups. The observed excellent early graft patency in combination with the limited outcome differences between grafting strategies makes the design of any future large RCT difficult. In the present pilot the lack of differences observed would suggest that an appropriately powered definitive study based on patency as an outcome would need to be prohibitively large. Our findings contrast with lower patency rates in saphenous vein grafts from older contemporary studies [12]. However, our findings of greater than 90% patency are remarkably similar to findings from recent trials in which graft patency was used as a primary outcome supporting our findings [13]. Taken together it is increasingly apparent that any benefit of TAG over conventional grafting may not manifest until much later in time making the design of a definitive study problematic. This is best illustrated by findings from large registries were the benefits increase with time but only become apparent after 10 years [2]. This is also apparent from findings from the ART trial comparing single IMA to bilateral IMA in which results at one year are comparable [14].

In the present study we chose to evaluate graft patency between groups with CT- Angiography based on its limited invasiveness, safety and reported accuracy in assessing graft patency [9]. However, we were surprised to report that significant difficulty in assessing graft patency may be an issue in up to 5% of grafts, particularly when evaluating sequential grafts on the lateral wall. Previous authors using the same scanner hardware and similar scanning protocols have reported varying results in terms of non – evaluable grafts ranging from 0% to 13% [15-18]. More recent studies using improved hardware and scanning protocols have also reported a small percentage of non-evaluable grafts [19]. In support of our observation is the observation by others that arterial grafts to the lateral wall may be particularly difficult to visualize [20]. Although the absolute numbers of non-diagnostic grafts are small, the low incidence rate of stenosis or occlusion calls into question the utility of CT-angio for the purpose of graft evaluation in this study population. Our findings appear to confirm that coronary angiography remains the gold standard for evaluation of coronary artery bypass grafts patency. More importantly for the design of endpoints in a trial comparing grafting strategies our observed difficulty with graft visualization would need to be taken into consideration for power calculations. One should note that CT technology is continuing to improve significantly such that this limitation may no longer be an issue in the future.

During the study period 421 patients were eligible for enrolment in the trial based on inclusion and exclusion criteria. However, only 60 patients (14.3%) were randomized and 2 of these patients withdrew. Recruitment was largely left to the participating surgeons making surgeon preference a major determinant of revascularization strategy. This explains why in this part of the manuscript we decided to explore clinical reasons why patients may not have been enrolled by creating a predictive model to determine the relative role of clinical variables in the decision making of surgeons to perform TAG outside of the trial. This approach was used to estimate the relative roles of surgeon preference as compared to objective clinical reasons for determining the use of TAG grafting. As one would expect patients who were older, required more urgent procedures and had more co-morbidities were least likely to undergo TAG. However, what is novel about our analysis is that surgeon was one of the most robust predictors of grafting strategy independent of all clinical variables. We interpret this finding as being highly suggestive that surgeons select patients for particular grafting strategy based on variables that are not captured by our database such as overall patient appearance, and also speculate that surgeon also use inherent biases about the relative benefit of one grafting strategy over another. Despite the above findings, we acknowledge that we remain unable to determine why exactly a particular patient was not enrolled.

## Conclusion

In summary, we have been able to demonstrate 3 important reasons why a trial comparing grafting strategy with TAG versus LIMA+SVG may be challenging. These reasons include a significant risk for slow enrolment, very small outcome differences between groups making clinically relevant conclusions difficult and limitations of using CT-angio as a tool to measure graft patency for the primary outcome. We therefore conclude that a larger multicenter study comparing graft patency of TAG vs. LIMA+SVG may not be feasible with the current design of the study in which early patency result would be the primary outcome. Our findings have important implications for the design of any future study in which grafting strategies are to be considered. The implications relate to the apparent limited differences in outcome differences early, limited patency differences early, methods by which grafts are assessed and inherent bias of surgeons making recruitment difficult. This means that longer-term clinical endpoints are likely most appropriate for the design of any future RCT to compare grafting strategies in cardiac surgery.

## Abbreviations

LIMA: Left internal mammary artery
RIMA: Right internal mammary artery
LAD: Left anterior descending artery
CABG: Coronary artery bypass graft surgery
TAG: Total arterial grafting
LIMA+SVG: LIMA to the LAD and saphenous vein graft

## References

[1] Elghobary T, Legare JF (2010). What has happened to multiple arterial grafting in coronary artery bypass grafting surgery?. Expert Rev Cardiovasc Ther.

[2] Kelly R, Buth KJ, Legare JF (2012). Bilateral internal thoracic artery grafting is superior to other forms of multiple arterial grafting in providing survival benefit after coronary bypass surgery. J Thorac Cardiovasc Surg.

[3] Lytle BW, Blackstone EH, Sabik JF, Houghtaling P, Loop FD, Cosgrove DM (2004). The effect of bilateral internal thoracic artery grafting on survival during 20 postoperative years. Ann Thorac Surg.

[4] Weiss AJ, Zhao S, Tian DH, Taggart DP, Yan TD (2013). A meta-analysis comparing bilateral internal mammary artery with left internal mammary artery for coronary artery bypass grafting. Ann Cardiothorac Surg.

[5] Guru V, Fremes SE, TU JV (2006). How many arterial grafts are enough? A population-based study of midterm outcomes. J Thorac Cardiovasc Surg.

[6] Tabata M, Grab JD, Khalpey Z, Edwards FH, O’brien SM, Cohn LH (2009). Prevalence and variability of internal mammary artery graft use in contemporary multivessel coronary artery bypass graft surgery: analysis of the Society of Thoracic Surgeons National Cardiac Database. Circulation.

[7] Muneretto C, Bisleri G, Negri A, Mandfredi J, Carone E, Morgan JA (2004). Left internal thoracic artery-radial artery composite grafts as the technique of choice for myocardial revascularization in elderly patients: a prospective randomized evaluation. J Thorac Cardiovasc Surg.

[8] Damgaard S, Wetterslev J, Lund JT, Lilleor NB, Perko MJ, Kelbaek H (2009). One-year results of total arterial revascularization vs. conventional coronary surgery: CARRPO trial. Eur Heart J.

[9] Abbara S, Arbab-Zadeh A, Callister TQ, Desai MY, Mamuya W, Thomson L (2009). SCCT guidelines for performance of coronary computed tomographic angiography: a report of the Society of Cardiovascular Computed Tomography Guidelines Committee. J Cardiovasc Comput Tomogr.

[10] Devereaux PJ, Chan MT, Alonso-Coello P, Walsh M, Berwanger O, Villar JC (2012). Association between postoperative troponin levels and 30-day mortality among patients undergoing noncardiac surgery. JAMA.

[11] Mohammed AA, Agnihotri AK, Arvind K, Van Kimmenade RR, Martinez-Rumayor A, Green SM (2009). Prospective, comprehensive assessment of cardiac troponin T testing after coronary artery bypass graft surgery. Circulation.

[12] Bourassa MG, Enjalbert M, Campeau L, Lesperance J (1984). Progression of atherosclerosis in coronary arteries and bypass grafts: ten years later. Am J Cardiol.

[13] Collins P, Webb CM, Chong CF, Moat NE (2008). Radial artery versus saphenous vein patency randomized trial: five-year angiographic follow-up. Circulation.

[14] Taggart DP, Altman DG, Gray AM, Lees B, Nugara F, Yu LM (2010). Randomized trial to compare bilateral vs. single internal mammary coronary artery bypass grafting: 1-year results of the Arterial Revascularisation Trial (ART). Eur Heart J.

[15] Malagutti P, Niemanm K, Meijboom WB, Van Mieghem CA, Pugliese F, Cademartiri F (2007). Use of 64-slice CT in symptomatic patients after coronary bypass surgery: evaluation of grafts and coronary arteries. Eur Heart J.

[16] Ropers D, Ulzheimer S, Wenkel E, Baum U, Giesler T, Derlien H (2001). Investigation of aortocoronary artery bypass grafts by multislice spiral computed tomography with electrocardiographic-gated image reconstruction. Am J Cardiol.

[17] Dikkers R, Van Der Zaag-Loonen HJ, Willems TP, Post WJ, Oudkerk M (2009). Is there an indication for computed tomography and magnetic resonance imaging in the evaluation of coronary artery bypass grafts?. J Comput Assist Tomogr.

[18] Jabara R, Chronos N, Klein L, Eisenburg S, Allen R, Bradford S (2007). Comparison of multidetector 64-slice computed tomographic angiography to coronary angiography to assess the patency of coronary artery bypass grafts. Am J Cardiol.

[19] Goetti R, Feuchtner G, Stolzmann P, Desbiolles L, Fishcer MA, Karlo C (2010). High-pitch dual-source CT coronary angiography: systolic data acquisition at high heart rates. Eur Radiol.

[20] Lee SK, Jung JI, Ko JM, Lee HG (2014). Image quality and radiation exposure of coronary CT angiography in patients after coronary artery bypass graft surgery: influence of imaging direction with 64-slice dual-source CT. J Cardiovasc Comput Tomogr.

